# Analysis of Superficial Subcutaneous Fat Camper’s and Scarpa’s Fascia in a United States Cohort

**DOI:** 10.3390/jcdd10080347

**Published:** 2023-08-14

**Authors:** David Z. Chen, Aravinda Ganapathy, Yash Nayak, Christopher Mejias, Grace L. Bishop, Vincent M. Mellnick, David H. Ballard

**Affiliations:** 1 School of Medicine, Washington University School of Medicine, St. Louis, MO 63110, USA; david.chen@wustl.edu (D.Z.C.); aganapathy@wustl.edu (A.G.); 2 School of Medicine, University of Michigan Medical School, Ann Arbor, MI 48109, USA; ynayak@med.umich.edu; 3 Mallinckrodt Institute of Radiology, Washington University School of Medicine, St. Louis, MO 63110, USA; christophermejias@wustl.edu (C.M.); bishop.g@wustl.edu (G.L.B.); mellnickv@wustl.edu (V.M.M.)

**Keywords:** Camper’s fascia, Scarpa’s fascia, visceral fascia

## Abstract

Together, the Camper’s and Scarpa’s fasciae form the superficial fat layer of the abdominal wall. Though they have clinical and surgical relevance, little is known about their role in body composition across diverse patient populations. This study aimed to determine the relationship between patient characteristics, including sex and body mass index, and the distribution of Camper’s and Scarpa’s fascial layers in the abdominal wall. A total of 458 patients’ abdominal CT examinations were segmented via CoreSlicer 1.0 to determine the surface area of each patient’s Camper’s, Scarpa’s, and visceral fascia layers. The reproducibility of segmentation was corroborated by an inter-rater analysis of segmented data for 20 randomly chosen patients divided between three study investigators. Pearson correlation and Student’s t-test analyses were performed to characterize the relationship between fascia distribution and demographic factors. The ratios of Camper’s fascia, both as a proportion of superficial fat (r = −0.44 and *p* < 0.0001) and as a proportion of total body fat (r = −0.34 and *p* < 0.0001), showed statistically significant negative correlations with BMI. In contrast, the ratios of Scarpa’s fascia, both as a proportion of superficial fat (r = 0.44 and *p* < 0.0001) and as a proportion of total body fat (r = 0.41 and *p* < 0.0001), exhibited statistically significant positive correlations with BMI. Between sexes, the females had a higher ratio of Scarpa’s facia to total body fat compared to the males (36.9% vs. 31% and *p* < 0.0001). The ICC values for the visceral fat, Scarpa fascia, and Camper fascia were 0.995, 0.991, and 0.995, respectively, which were all within the ‘almost perfect’ range (ICC = 0.81–1.00). These findings contribute novel insights by revealing that as BMI increases the proportion of Camper’s fascia decreases, while the ratio of Scarpa’s fascia increases. Such insights expand the scope of body composition studies, which typically focus solely on superficial and visceral fat ratios.

## 1. Introduction

In the current literature, the Camper and Scarpa fasciae are collectively described as components of the “superficial fascia” of the abdominal wall, located directly under the skin and superficial adipose layers. Camper’s fascia is the outer fatty layer while Scarpa’s fascia is the deeper membranous layer below Camper’s [1]. Both fasciae are noted to have important clinical and surgical relevance. The fasciae work together to insulate deep abdominal organs from integument, absorb and attenuate force, and regulate body temperature [1,2]. Additionally, they protect and facilitate the sensory functions of intercostal and subcostal nerves [1,2]. Surgically, Camper’s fascia is considered an important landmark for inguinal hernia repair, and surgeons often approximate this layer with abdominal wall closures [3]. Mass closure of the space between Camper’s and Scarpa’s fascia is associated with lower recurrence of incisional herniation and seroma formation [4]. Previous studies have highlighted the importance of Scarpa’s fascia preservation in preventing seroma formation, reducing total drain output, and length of hospital stay following abdominoplasty procedures [5,6]. Surgical techniques involving manipulation and preservation of the Scarpa fascia have also been used to improve the aesthetics of flaps harvested from the abdominal wall subcutaneous fat [7,8].

**Figure 1:**
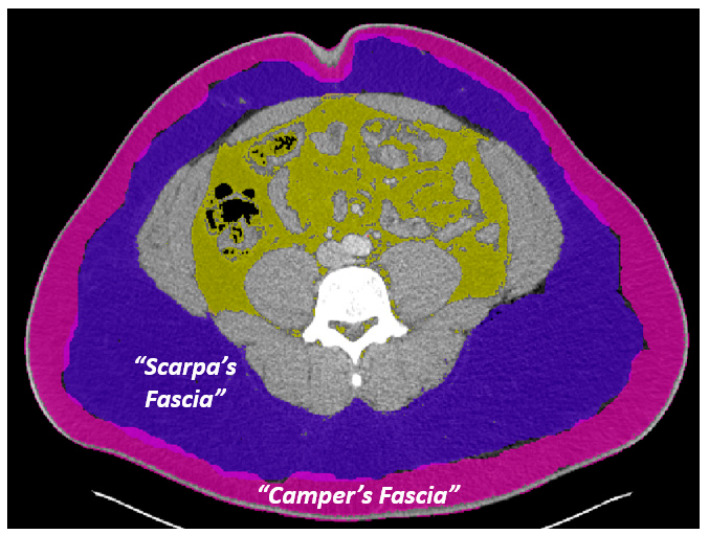
Superficial Fascia Layer Naming Conventions. Camper’s fascia (pink overlay) and Scarpa’s fascia (purple overlay) are labeled, whereas the yellow overlay denotes the visceral fat.

Notably, there has been some controversy over the existence of Camper’s and Scarpa’s fasciae and the distribution of the superficial fascia. Some authors challenge the existence of Scarpa’s fascia altogether, describing only a single layer of superficial fascia [9,10]. In addition, to make matters more complicated, while Camper’s and Scarpa’s fasciae have typically been portrayed as a single superficial layer (Camper’s) overlying a membranous layer (Scarpa’s), in reality there appears to be an additional fatty layer underneath the membranous layer [11,12,13]. Select studies within the diabetes and metabolism literature have referred to what is traditionally understood to be “Camper’s fascia” as the “superficial subcutaneous adipose tissue” (SSAT), the membranous layer as “Scarpa’s fascia”, and the deeper fat layer as “deep subcutaneous adipose tissue” (DSAT) [14,15]. Other studies have referred to the deeper fatty layer as “sub-Scarpal fat” [16]. For the purposes of this study, we will refer to the more superficial fatty layer as “Camper’s fascia” and the deeper fatty layer as “Scarpa’s fascia” (Figure 1).

In addition to these ongoing discussions, the fascial layers still have many properties yet to be fully assessed. These properties include the potential for regeneration in wound repair and potential reduction in surgical complications when preserving Scarpa fascia [1,17,18,19,20]. Lancerotto et al. performed histological and radiological measurements of the two fasciae, which they dubbed the “membranous layer”, and noted discrepancies in thickness between measurements made on histology and CT images [21]. They additionally noted the ratio of the membranous layer to the surrounding fat layers varied between slim and obese patients [21]. This issue proves to be problematic as fasciae are often depicted in a monolithic fashion by anatomical guides and resources.

Given their clinical importance and wide surgical utility, these fasciae should be investigated further and better characterized. The current literature supports the notion that a patient’s visceral fat to body mass ratio is indicative of several long-term health outcomes [22,23,24]. With regard to the superficial fascia, there exists a body of literature dating back to 2001 in the diabetes and metabolism space which was not initially discovered by our literature search as it describes the separate fascial compartments as “superficial subcutaneous adipose tissue” (Camper’s fascia) and “deep subcutaneous adipose tissue” (Scarpa’s fascia) [25]. Additional studies have characterized the relationship between superficial adipose distribution and metabolic markers including fasting insulin, Hb A1c, and glycemic profile [11,25,26]. Further characterizing differences in abdominal fascia composition between different patient populations may lend insight about the association between superficial subcutaneous fat and long-term health. Frank et al. previously performed a study regarding the variance in thickness of abdominal wall layers across age and BMI; however, they focused solely on Scarpa’s fascia and used fascial thickness as their variable of interest [11]. Due to its broad significance in both clinical and surgical settings, a better understanding of both Camper’s and Scarpa’s fasciae may offer pertinent information impacting overall patient treatment outcomes. In this study, we aimed to characterize the relationship between patient characteristics, including body mass index and sex, and the distribution of Camper’s and Scarpa’s fascial layers in the abdominal wall in an American cohort.

## 2. Materials and Methods

This was a single-center Institutional Review Board—approved Health Insurance Portability and Accountability Act—compliant study. We retrospectively identified patients from a prospectively maintained trauma database during a 5-year study period, 17 November 2014, to 26 November 2019. Inclusion criteria included patients who underwent a CT (abdomen and pelvis) in the initial evaluation of trauma and had adequate CT imaging for body composition analysis as assessed by study investigators. Exclusion criteria included patients who did not receive imaging or had surgical management prior to initial CT imaging. Basic demographic information from each patient was collected. We did not assess trauma outcomes as it was outside the scope of the study.

CT acquisitions included CT examinations of the abdomen and pelvis with contrast. CTs were performed on a number of different generation Siemens scanners over the 5-year study period. For composition analysis, CT examinations that covered the area from the umbilicus with less than 10% collimation of the abdominal wall within the field of view were considered adequate for accurate quantification. Patients with insufficient field of view, with more than 10% collimation of the body wall at the umbilical level, were excluded from this analysis to ensure reliable fat quantification. We accepted some degree of collimation as this captured patients with high BMI.

**Figure 2:**
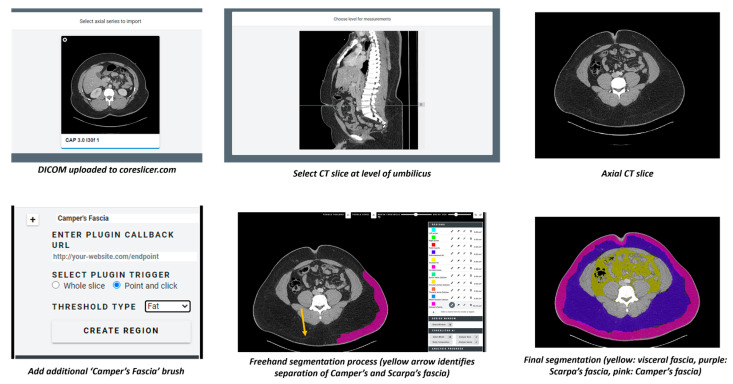
CoreSlicer Segmentation Process. The yellow arrow denotes the separation between the Camper’s and Scarpa’s fasciae.

Segmentation of CT scans for all patients in the study was performed by a radiology resident supervised by a fellowship trained abdominal radiologist using CoreSlicer version 1.0 at www.coreslicer.com (accessed on 16 September 2022). For each patient, depersonalized DICOM files were uploaded to CoreSlicer. The desired segmentation slice was chosen by navigating to the umbilicus in the sagittal view. Segmentation then proceeded on the corresponding axial slice of the umbilicus. Three different layer masks were then created and designated for visceral, Scarpa, and Camper’s fasciae (CoreSlicer automatically generates layer masks for superficial and visceral fat, but delineation of superficial fat into Camper’s and Scarpa’s fasciae required manual addition of a custom layer mask). Regions for each of the three fasciae were then grown freehand under their respective masking layer. Surface area of each fascia layer was automatically calculated using the three different CoreSlicer masking layers. The entire segmentation process can be visualized in Figure 2.

Inter-rater reliability analysis was conducted to determine whether segmentation values obtained by the radiology resident were reproducible. Two study investigator non-radiologists, one with basic medical training and one with no medical training, used CoreSlicer version 1.0 to segment 20 randomly chosen patients from the study with representative heterogeneity. They each independently performed the segmentation protocol described above to obtain surface area values for visceral, Scarpa, and Camper’s fasciae of the 20 patients. RStudio version 2021.09.2 was used to calculate inter-rater reliability. Agreement levels were evaluated using the intraclass correlation coefficient (ICC) for continuous variables. Agreement was classified as poor (ICC < 0.00), slight (ICC = 0.00–0.20), fair (ICC = 0.21–0.40), moderate (ICC = 0.41–0.60), substantial (ICC = 0.61–0.80), or almost perfect (ICC = 0.81–1.00).

Statistical analysis was performed using GraphPad Prism version 8.0.0 for Windows, GraphPad Software, San Diego, CA, USA, www.graphpad.com (15 March 2023). Pearson’s test of correlation was performed to evaluate the relationship between distribution of superficial fasciae and BMI. Pearson correlation was reported as r (−1.0 = perfect negative correlation, 1.0 perfect positive correlation, and 0 = no correlation). Student’s t-test was performed to analyze the correlation between superficial fascial distribution and demographic characteristics. *p* < 0.05 was considered statistically significant.

## 3. Results

### 3.1. Patient Characteristics

**Figure 3:**
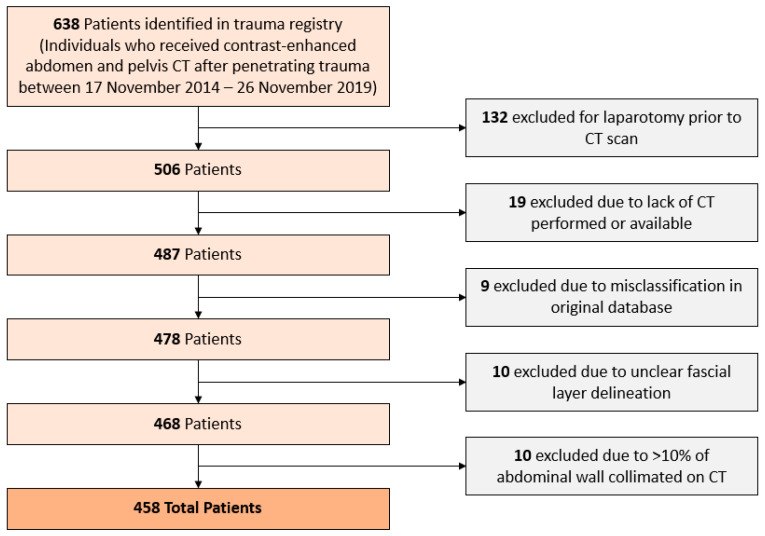
Study Population Exclusion Criteria.

Our initial sample contained a total of 638 patients identified from the trauma registry. A total of 132 patients were excluded as they received laparotomy prior to an initial CT scan, with postsurgical changes potentially interfering with fat segmentation. Patients who had no CT performed or available (*n* = 19), patients who were misclassified in the original database (*n* = 9), patients whose fascial layers could not be clearly differentiated (*n* = 10), and patients who had >10% of abdominal wall collimated on CT (*n* = 10) were excluded from analysis. A summary of these exclusion criteria is included in Figure 3. A total of 458 patients were included in the final analysis; demographic information and indications for CT are presented in Table 1. Of note, ‘Total Fat’ refers to the sum of a patient’s Camper’s fascia, Scarpa’s fascia, and visceral fascia, while ‘Superficial Fat’ refers to the sum of a patient’s Camper’s fascia and Scarpa’s fascia. Accordingly, ‘Total Ratio’ refers to the proportion of total fat made up by either the Camper’s or the Scarpa’s fascia, and ‘Superficial Ratio’ refers to the proportion of superficial fat made up by either the Camper’s or the Scarpa’s fascia.

**Figure 4:**
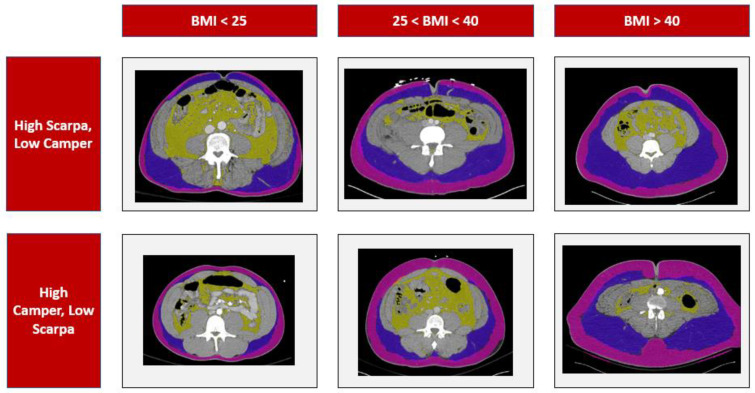
Case examples demonstrating different distributions of superficial fat across BMI categories. Pink overlay represents Camper’s fascia, purple overlay represents Scarpa’s fascia, and yellow overlay represents visceral fat.

Our sample demonstrated examples of the diverse distributions of the superficial fat within individuals, with different patients demonstrating a higher proportion of Camper’s fascia, a higher proportion of Scarpa’s fascia, or a relatively even distribution, regardless of their BMI. These findings are demonstrated in Figure 4.

### 3.2. Group Comparisons

**Figure 5:**
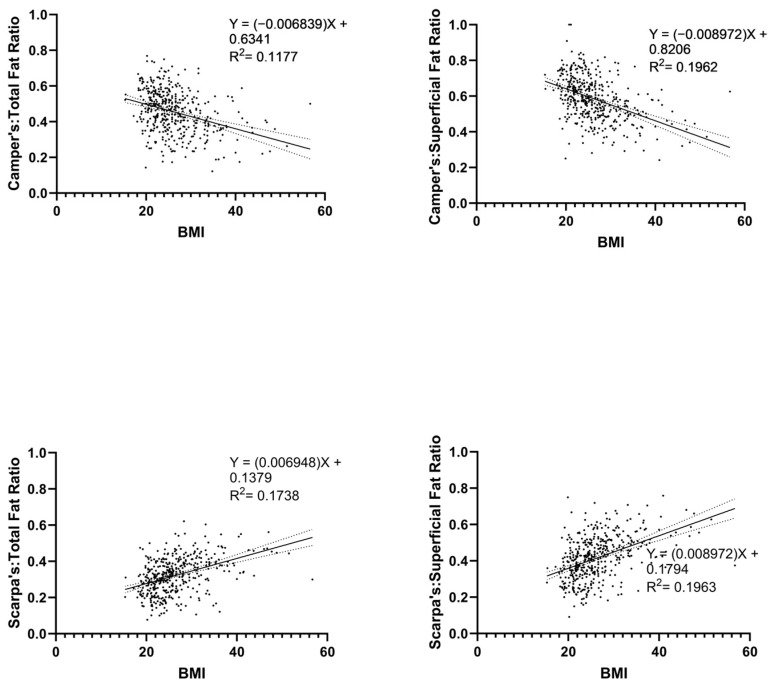
Correlational Findings of BMI with Camper’s and Scarpa’s Ratios. ‘Total Fat’ refers to the sum of a patient’s Camper’s fascia, Scarpa’s fascia, and visceral fascia, while ‘Superficial Fat’ refers to the sum of a patient’s Camper’s fascia and Scarpa’s fascia. Accordingly, ‘Total Ratio’ refers to the proportion of total fat made up by either Camper’s or Scarpa’s fascia, and ‘Superficial Ratio’ refers to the proportion of superficial fat made up by either Camper’s or Scarpa’s fascia Solid and dotted lines show mean and error bars, respectively.

The ratios of Camper’s fascia, both as a proportion of superficial fat (r = −0.44 and *p* < 0.0001) and as a proportion of total body fat (r = −0.34 and *p* < 0.0001), showed statistically significant negative correlations with BMI. In contrast, the ratios of Scarpa’s fascia, both as a proportion of superficial fat (r = 0.44 and *p* < 0.0001) and as a proportion of total body fat (r = 0.41 and *p* < 0.0001), exhibited statistically significant positive correlations with BMI. These correlation findings are depicted in Figure 5.

**Figure 6:**
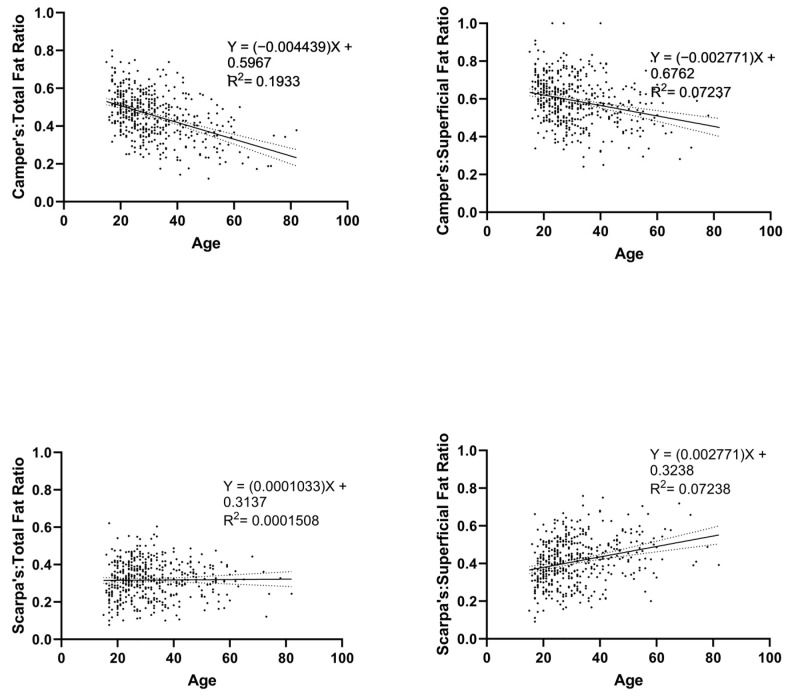
Correlational Findings of Age with Camper’s and Scarpa’s Ratios. ‘Total Fat’ refers to the sum of a patient’s Camper’s fascia, Scarpa’s fascia, and visceral fascia, while ‘Superficial Fat’ refers to the sum of a patient’s Camper’s fascia and Scarpa’s fascia. Accordingly, ‘Total Ratio’ refers to the proportion of total fat made up by either Camper’s or Scarpa’s fascia, and ‘Superficial Ratio’ refers to the proportion of superficial fat made up by either Camper’s or Scarpa’s fascia. Solid and dotted lines show mean and error bars, respectively.

Age was negatively associated with a ratio of Camper’s fascia to total fat (r = −0.44 and *p* < 0.0001) and also negatively associated with a ratio of Camper’s fascia to superficial fat (r = −0.27 and *p* < 0.0001). Age was not associated with the ratio of Scarpa’s fascia as a proportion of total fat (r = 0.012 and *p* = 0.7932) but was positively associated with the ratio of Scarpa’s fascia to superficial fat ratio (r = 0.27 and *p* < 0.0001). These correlation findings are depicted in Figure 6.

Female sex was associated with a higher ratio of Scarpa’s fascia as a proportion of total body fat compared to male sex (36.9% vs. 31% and *p* < *0*.0001) but was not associated with any significant difference in the ratio of Camper’s fascia to total (48.6% vs. 45.3% and *p* = 0.0656) or superficial fat (56.7% vs. 59.2% and *p* = 0.1602). Results by sex are demonstrated in Table 2.

### 3.3. Inter-Rater Reliability

**Figure 7:**
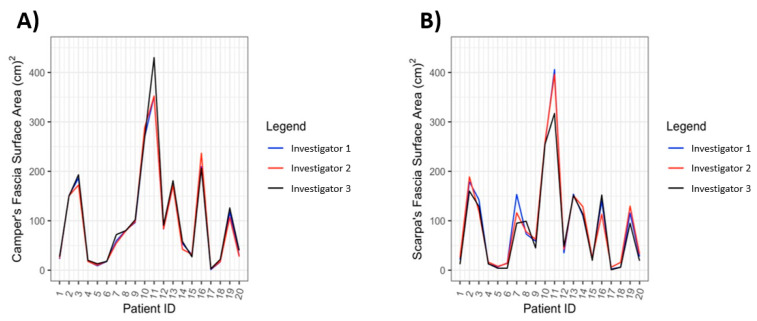
Inter-rater Variation within Camper’s Fascia Surface Area Measurements (**A**) and Scarpa’s Fascia Surface Area Measurements (**B**).

The ICC values for the visceral fat, Scarpa fascia, and Camper fascia were 0.995, 0.991, and 0.995, respectively, which were all within the ‘almost perfect’ range (ICC = 0.81–1.00). To visualize the variation in surface area measurements amongst the raters, data collected by all three raters are plotted amongst each other for each respective fascia (Figure 7A,B).

## 4. Discussion

### 4.1. Study Results

In this study we aimed to better characterize the abdominal wall subcutaneous fat Camper’s and Scarpa’s fasciae within patients compared to BMI. As noted previously, some aspects of these fascia layers, such as their potential roles in surgical considerations beyond abdominoplasty, are yet to be fully assessed [17,18,19,22,23]. To the best of our knowledge based on our literature search, this is the first study demonstrating that patient age and BMI are associated with a greater proportion of Scarpa’s fascia, suggesting that the body may preferentially store fat in these areas with age or increased weight gain. Although previous studies have established a link between metabolic syndrome and higher levels of visceral adipose tissue [27], there remains a knowledge gap regarding the proportions of the Camper’s and the Scarpa’s fasciae in this context. Understanding the role of these fascial proportions could contribute to understanding metabolic syndrome’s pathophysiology and its risk factors. The current study provides novel insights by demonstrating that as BMI increases, the proportion of Camper’s fascia decreases while the proportion of Scarpa’s fascia increases. These findings offer additional insights for body composition studies that focus solely on superficial and visceral fat ratios. Only one study using a United States cohort has demonstrated a relationship between deep subcutaneous abdominal adipose tissue and fasting insulin levels after adjusting for total body fat [14].

Body composition studies assessing superficial and visceral fat proportions commonly employ automatic or semiautomated software, typically validated by a study investigator. In our study, semiautomated software was utilized initially, followed by manual tracings to determine the measurements of Camper’s and Scarpa’s fasciae. Our inter-rater reliability analysis demonstrated a strong, nearly perfect agreement among the three raters’ measurements for Camper’s and Scarpa’s fasciae. These findings indicate that manual segmentation with CoreSlicer is a reliable and precise method for quantifying Camper’s and Scarpa’s fasciae in body composition studies. The consistency observed in our results highlights the reliability and adaptability of the manual segmentation process employed with CoreSlicer to quantify Camper’s and Scarpa’s fasciae. This reliability remains consistent regardless of the operator’s level of training. The strong agreement observed also suggests the potential for incorporating machine learning models into body composition analyses to quantify these advanced levels of superficial fat. These findings open avenues for further exploration and the development of advanced techniques in body composition assessment.

Fascial composition variation is likely the result of both environmental factors such as activity and molecular factors such as adiponectin and TNF-α. Circulating adiponectin and TNF-α levels have been inversely correlated with superficial, deep, and visceral adipose tissue composition [13]. Furthermore, increased resting energy expenditure has also been associated with decreased abdominal superficial subcutaneous adipose tissue and increased visceral adipose tissue proportion [28]. These molecular and environmental differences leading to compositional changes can act as proxies for potential risk quantification. Body composition analysis via imaging provides a useful measurable indicator of risk for cardiovascular disease, as demonstrated by various studies using various cohorts [29,30,31,32]. Use of BMI alone to determine cardiometabolic risk may misclassify patients who would otherwise be correctly classified if a body composition analysis were used [31]. Our purpose in conducting this study was to demonstrate that body composition not only varies by general adiposity but also by fascial distribution, which can be analyzed using imaging and reconstruction. These compositional fascial differences may serve as early indicators for cardiometabolic risk and other clinical outcomes.

Further studies should focus on outcomes research linking compositional differences within individuals to cardiometabolic or cardiovascular disease. It remains to be seen how the distribution of superficial and deep subcutaneous tissue predisposes individuals to cardiovascular disease, and it remains to be seen whether alterations in fascial composition can help prevent or limit risk of developing such diseases. Finally, these studies should correlate to other known risk factors for cardiovascular disease and metabolic syndrome; this will allow optimal quantification of future risk.

### 4.2. Study Limitations

The limitations of this study include the retrospective nature and the sample being limited to a single institution. In addition, the study cohort consisted primarily of patients presenting for trauma indications. The overall sample consisted of 458 patients, and a large majority of this sample was male (~85%) and/or black (~85%). Given these demographic characteristics, the overall study population may not be representative of the general population. While the study results were statistically significant, future studies should aim to recruit a significantly larger population with a more even distribution of demographic characteristics in order to ensure broader generalizability and further validate these study findings. The purpose of the design of our convenience sample cohort was primarily to maximize the inclusion of patients with CT examinations.

In addition, although we were able to find compositional differences within patients, future studies should aim to characterize the relationship between these differences to clinical outcomes. While our study analyzed differences in proportions of subcutaneous fat layers as BMI changes, future imaging-based studies could be designed to discern subtle compositional differences between the Scarpa’s and the Camper’s fascia. This would likely require analysis of larger, segmented volumes of interest—such as the entire abdominal wall—rather than a single slice, and the application of texture analysis and feature extraction radiomics analyses.

## 5. Conclusions

Fascial compositional differences in both superficial and deep subcutaneous tissue exist within patients. Our study is the first to demonstrate increased proportion of Scarpa’s fascia as a function of BMI along with a decreased proportion of Camper’s fascia relative to BMI. Future study designs should recruit a broader, more representative study population and focus on correlating these compositional differences with cardiometabolic disease outcomes so that an optimal quantification of cardiometabolic risk can be obtained utilizing body compositional analysis.

## Figures and Tables

**Table 1:** Demographics and Mean Values of Patients Included in Study. ‘Total Fat’ refers to the sum of a patient’s Camper’s fascia, Scarpa’s fascia, and visceral fascia, while ‘Superficial Fat’ refers to the sum of a patient’s Camper’s fascia and Scarpa’s fascia. Accordingly, ‘Total Ratio’ refers to the proportion of total fat made up by either Camper’s or Scarpa’s fascia, and ‘Superficial Ratio’ refers to the proportion of superficial fat made up by either Camper’s or Scarpa’s fascia.

Demographics	Female	Male	Total
n	53	405	458
Race, n (%)			
Asian	0 (0%)	4 (1%)	4 (0.9%)
Black	43 (81.1%)	346 (85.4%)	389 (84.9%)
Other	0 (0%)	5 (1.2%)	5 (1.1%)
Unknown	2 (3.8%)	10 (2.5%)	12 (2.6%)
White	8 (15.1%)	40 (9.9%)	48 (10.5%)
Age, mean ± SD (min–max)	31.2 ± 11.7 (16–78)	31.5 ± 12 (15–82)	31.5 ± 12 (15–82)
BMI, mean ± SD (min–max)	28.4 ± 7.18 (18.3–47.7)	25.8 ± 5.66 (15.3–56.7)	26.1 ± 0.28 (15.3–56.7)
Total Fat, mean ± SD (min–max)	423 ± 237 (46–1080)	214 ± 210(4–1080)	239 ± 224 (4–1080)
Superficial Fat, mean ± SD (min–max)	357 ± 199 (39–944)	163 ± 162 (2–763)	185 ± 178 (2–944)
Camper’s Fascia, mean ± SD (min–max)	192 ± 97 (27–438)	84 ± 75 (1–449)	96.6 ± 85.2 (1–449)
Camper’s Total Ratio, mean ± SD (min–max)	0.487 ± 0.103 (0.262–0.698)	0.455 ± 0.123 (0.122–0.8)	0.459 ± 0.121(0.122–0.8)
Camper’s Superficial Ratio, mean ± SD (min–max)	0.567 ± 0.0955 (0.338–0.784)	0.593 ± 0.127 (0.241–1)	0.59 ± 0.124 (0.241–1)
Scarpa’s Fascia, mean ± SD (min–max)	165 ± 113 (12–506)	78.5 ± 93.1 (0–479)	89.3 ± 4.6 (0–506)
Scarpa’s Total Ratio, mean ± SD (min–max)	0.369 ± 0.0853 (0.185–0.621)	0.31 ± 0.101 (0–0.604)	0.317 ± 0.101 (0–0.621)
Scarpa’s Superficial Ratio, mean ± SD (min–max)	0.433 ± 0.0955 (0.216–0.662)	0.407 ± 0.127 (0–0.759)	0.411 ± 0.124 (0–0.759)
Visceral Fat, mean ± SD (min–max)	66 ± 56.3 (4–205)	51.6 ± 60.2 (1–316)	53.3 ± 59.9 (1–316)
Visceral Total Ratio, mean ± SD (min–max)	0.144 ± 0.0879 (0.03–0.376)	0.234 ± 0.116 (0.036–0.723)	0.224 ± 0.117 (0.03–0.723)

**Table 2:** Results Table by Sex. ‘Total Fat’ refers to the sum of a patient’s Camper’s fascia, Scarpa’s fascia, and visceral fascia, while ‘Superficial Fat’ refers to the sum of a patient’s Camper’s fascia and Scarpa’s fascia. Accordingly, ‘Total Ratio’ refers to the proportion of total fat made up by either Camper’s or Scarpa’s fascia, and ‘Superficial Ratio’ refers to the proportion of superficial fat made up by either Camper’s or Scarpa’s fascia.

Camper’s: Total Fat Ratio	Female	Male
**Mean**	0.486	0.453
**SD**	0.1022	0.1226
***p*-value**	0.0656	
**95% CI of Difference**	−0.002107 to 0.06704	
**Camper’s: Superficial Fat Ratio**	Female	Male
**Mean**	0.5666	0.5919
**SD**	0.09515	0.1262
***p*-value**	0.1602	
**95% CI**	−0.06061 to 0.01004	
**Scarpa’s: Total Fat Ratio**	Female	Male
**Mean**	0.3692	0.3101
**SD**	0.08511	0.1006
***p*-value**	<0.0001	
**95% CI of Difference**	0.03067 to 0.08747	
**Scarpa’s: Superficial Fat Ratio**	Female	Male
**Mean**	0.4334	0.4081
**SD**	0.09515	0.1262
***p*-value**	0.1602	
**95% CI of Difference**	−0.01004 to 0.06060	

## Data Availability

The data presented in this study are available on request from the corresponding author. The data are not publicly available due to patient information.
